# Optical characteristics of human lung cancer for photodynamic therapy with measured absorption and reduced scattering coefficients

**DOI:** 10.1117/1.JBO.30.4.048001

**Published:** 2025-04-01

**Authors:** Yu Shimojo, Yuri Morizane, Takumi Sonokawa, Jitsuo Usuda, Takahiro Nishimura

**Affiliations:** a Osaka University, Graduate School of Engineering, Osaka, Japan; b Osaka Metropolitan University, Graduate School of Medicine, Osaka, Japan; c Research Fellow of Japan Society for the Promotion of Science, Tokyo, Japan; d Nippon Medical School Hospital, Department of Thoracic Surgery, Tokyo, Japan

**Keywords:** lung, optical properties, light distribution, human tissue, photodynamic therapy, peripheral lung cancer

## Abstract

**Significance:**

The optical characteristics of a human lung, such as the light distribution in the tissue, are crucial for evaluating the light delivery of photodynamic therapy (PDT) for peripheral lung cancer.

**Aim:**

The light distribution in the human lung is analyzed with absorption ($\mu_{a}$) and reduced scattering ($\mu_{s}^{\prime }$) coefficients measured *ex vivo* for normal, carbon-deposited, and tumor tissues.

**Approach:**

The $\mu_{a}$ and $\mu_{s}^{\prime }$ spectra were measured using a double-integrating-sphere optical system and inverse Monte Carlo technique. The measured values were used to perform a light distribution analysis using a Monte Carlo light transport simulation.

**Results:**

The $\mu_{a}$ values varied between tissue types owing to the influence of carbon deposition, blood volume fraction, and oxygen saturation, whereas the $\mu_{s}^{\prime }$ values showed almost no differences between tissue types. The simulation results showed that carbon deposition in the surrounding tissue and oxygen saturation variability had almost no effect on PDT light delivery to a tumor with a 10-mm-diameter sphere.

**Conclusions:**

Our analysis revealed the influence of the optical characteristics of the lung tissue on PDT light delivery. Integration of these results with the photosensitizer dose and the degree of necrosis changes will allow us to provide more clinically relevant insight in determining PDT dosimetry.

## 1 Introduction

Lung cancer remains the leading cause of cancer-related mortality,^1^ with ∼60% of lung cancers arising in the peripheral regions.^2^ Recent developments in bronchoscopy and navigation technologies have significantly improved the diagnostic accuracy for these tumors.^3^^,^^4^ Standard treatment for peripheral lung cancer typically involves a lobectomy or sublobar resection.^5^ However, surgery may not be feasible in elderly patients owing to comorbidities such as emphysema or interstitial pneumonia. Although there are clinical reports of percutaneous radiofrequency and microwave ablation, these procedures risk complications such as pneumothorax.^6^^–^^8^ Therefore, there is growing interest in photodynamic therapy (PDT) for peripheral lung cancer.^9^ PDT shows its antitumor effects by generating singlet oxygen through the interaction of laser light with a photosensitizer accumulated in the target tissue. In thoracic surgery, PDT using talaporfin sodium as a photosensitizer has demonstrated favorable outcomes for centrally located early-stage lung cancer.^10^^–^^12^ For peripheral lung cancer, interstitial PDT has been studied, in which light is delivered to tumors through fibers guided by needles inserted percutaneously under image guidance.^13^ Recently, the use of thin optical probes has enabled the safe delivery of laser light to narrow areas through a bronchoscopic approach, leading to clinical trials aimed at applying this type of PDT to peripheral lung cancer.^14^ Because laser irradiation triggers the photochemical reaction in both percutaneous and bronchoscopic PDT, understanding the light distribution in the tumor tissue is essential for assessing the safety and efficacy of PDT for peripheral lung cancer.

The light distribution in the lung can be obtained using the absorption coefficient ($\mu_{a}$) and reduced scattering coefficient ($\mu_{s}^{\prime }$) in computational methods such as Monte Carlo (MC) simulations and diffusion approximations.^15^^,^^16^ The accuracy of these physical parameters affects the simulation results. Optical properties of human lung tissue have been measured by *ex vivo* and *in vivo* diffuse reflectance spectroscopy. However, *ex vivo* data are limited to specific wavelengths (406 to 415, 515, 632.8, 635, and 676.4 nm)^17^^–^^19^ and cannot be used to calculate the light distribution at 664 nm, which is used in talaporfin sodium PDT for lung cancer.^10^^–^^12^ Another study endoscopically measured *in vivo* optical properties in the wavelength range 480 to 925 nm,^20^ but the limited number of patients (n=2) makes it difficult to analyze the differences between normal and tumor tissues. Moreover, variations in carbon deposition among patients are expected to cause variability in optical properties. These aspects highlight the need to measure the $\mu_{a}$ and $\mu_{s}^{\prime }$ spectra of lung tissues from a large number of patients and to evaluate the impact of the differences between different tissue types on laser irradiation.

In this study, we analyze the optical characteristics of human lung tissue, such as the light distribution in the tissue, to evaluate the light delivery of PDT for peripheral lung cancer. The optical properties of normal, carbon-deposited, and tumor tissues are measured using a double-integrating-sphere (DIS) optical system and the inverse Monte Carlo (IMC) method. Optical properties of normal and tumor tissues in human lungs are obtained by comparing measured values of diffuse reflectance ($R_{d}$) and total transmittance ($T_{t}$) with IMC-based calculations. Furthermore, similar measurements are performed for porcine lung tissue because porcine lung models have the potential to be used for preclinical evaluation. The measured values are used to calculate the light distribution in the lung with MC simulations. These simulations allow assessment of the effects of carbon deposition and blood oxygen saturation, and the differences between human and porcine tissues on light delivery for PDT. These results are expected to provide a critical and quantitative understanding of light propagation in the human lung when irradiated with an optical probe for PDT of peripheral lung cancer.

## 2 Materials and Methods

### 2.1 Sample Preparation

The study was conducted in accordance with the protocol approved by the Central Ethics Committee of the Nippon Medical School Foundation (approval number: 2023-1067), and informed consent was obtained from all participating patients. Tissue samples were obtained from operations performed at the Nippon Medical School Hospital. Table 1 provides detailed information on the patients and their respective samples. A total of nine patients (eight males and one female) with a mean age of 70±17 years were enrolled in the study. The resected tissues included normal tissue, carbon-deposited tissue, and tumor tissue, and measurements were performed within 5 h of resection. Normal and carbon-deposited tissues were distinguished by whether a light spot was produced on the site where the carbon powder had adhered. The resected tissues were deflated. They were stored in saline-moistened gauze until measurement. To prepare the samples, the thicknesses of the normal and tumor tissues were adjusted with scissors. Sample thickness was measured three times with a micrometer (MDC-25PX, Mitutoyo, Kanagawa, Japan), and the average value was recorded. The sample thickness ranged from 0.4 to 0.9 mm for normal tissues, was 0.7 mm for carbon-deposited tissues, and ranged from 0.8 to 1.1 mm for tumor tissues. The measured thickness of each sample was used for the IMC analysis. Their thicknesses were fixed with multiple stainless steel spacers (0.1, 0.5, and 1.0 mm) and sandwiched between glass slides to prevent sample compression. At this time, saline drops were applied to the tissues, and they were sealed with parafilm to prevent dehydration. For porcine samples, normal lung tissue was procured from Tokyo Shibaura Zhoki, Tokyo, Japan. The preparation procedure for porcine samples was identical to that for human samples. Sample thickness ranged from 0.5 to 0.8 mm.

**Table 1:** Information on patients and samples.

ID	Sex	Age	Site^a^	Tumor type	Sample^b^
#1	Male	47	LUL	—	N
#2	Male	83	RUL	Adenocarcinoma	N, C, T
#3	Male	83	RLL	Adenocarcinoma	N, C, T
#4	Male	63	LUL	Adenocarcinoma	N, T
#5	Male	37	RUL	Adenocarcinoma	N
#6	Male	76	LLL	Adenocarcinoma	N, T
#7	Male	85	LLL	Squamous cell carcinoma	N, T
#8	Male	74	LUL	Combined small cell carcinoma	N, T
#9	Female	79	RLL	Squamous cell carcinoma	N, T

a LLL: left lower lobe; LUL: left upper lobe; RLL: right lower lobe; RUL: right upper lobe.

b C: carbon-deposited tissue; N: normal tissue; T: tumor tissue.

### 2.2 Measurement of Absorption and Reduced Scattering Coefficients

The $\mu_{a}$ and $\mu_{s}^{\prime }$ spectra were obtained using a DIS optical system and the IMC method. The procedures have been previously described in detail elsewhere.^21^ A xenon lamp (XEF152-S, Kenko Tokina, Tokyo, Japan) was used as the light source. White light emitted from the light guide was focused on a 1-mm spot at the sample position. The reflected and transmitted light from the sample was detected using integrating spheres (4P-GPS-033-SL, Labsphere, North Sutton, New Hampshire, United States) and an optical fiber (P600-1-VIS-NIR, Ocean Insight, Orlando, Florida, United States) connected to a spectrophotometer (Maya2000Pro, Ocean Insight). The port diameter of the integrating spheres was 0.25 in. The exposure time was set to 140 ms, and measurements were averaged over 100 scans. Each measurement was repeated three times and averaged. All measurements were performed at room temperature in a dark room. The measurement accuracy for $R_{d}$ and $T_{t}$ was within 0.8% in the wavelength range 400 to 800 nm.^21^ The $\mu_{a}$ and $\mu_{s}^{\prime }$ spectra were calculated from the measured $R_{d}$ and $T_{t}$ values using the IMC method.^21^ For this analysis, the refractive index n was set to 1.38, which was based on literature values for lung tissue,^22^ and the anisotropy factor g was set to 0.9 because this value is typical for many tissues in the wavelength range used.^23^

### 2.3 Statistical Analysis

The optical properties of normal tissue versus carbon-deposited tissue and normal tissue versus tumor tissue were evaluated for statistical significance using the Student unpaired two-tailed t-test. A p-value of <0.01 was considered indicative of statistical significance. p-values were plotted against the wavelength to identify wavelength ranges with significant differences in optical properties. Experimental data were expressed as mean ± standard deviation.

### 2.4 Analysis of Oxygen Saturation Variation During Measurement

In *ex vivo* experiments, the blood oxygen saturation may vary during measurements. To evaluate this, oxygen saturation was determined from the measured $\mu_{a}$ spectra of normal and tumor human tissues. This analysis assumes that the absorption coefficient can be fully approximated using a linear combination of chromophore spectra.^15^ The absorption coefficient of the tissue was modeled as a linear combination of the coefficients of water ($\mu_{a}^{\text{water}}$),^24^ dry bloodless tissue (background, $\mu_{a}^{\text{dry}}$), oxyhemoglobin ($\mu_{a}^{\mathrm{oxy}}$),^25^ and deoxyhemoglobin ($\mu_{a}^{\mathrm{deoxy}}$),^25^ as expressed by the following equation:^20^

$$\mu_{a}(\lambda )=\mu_{a}^{\text{dry}}(\lambda )+f_{w}\mu_{a}^{\text{water}}(\lambda )+f_{b}[{\mathrm{SO}}_{2}\mu_{a}^{\mathrm{oxy}}(\lambda )+(1-{\mathrm{SO}}_{2})\mu_{a}^{\mathrm{deoxy}}(\lambda )],$$ (1)

$$\mu_{a}^{\text{dry}}(\lambda )=A\;\exp(-B\lambda ),$$ (2)

where λ is the wavelength, $f_{b}$ is the blood volume fraction, ${\mathrm{SO}}_{2}$ is the oxygen saturation, and $f_{w}$ is the water volume fraction. The absorption of dry bloodless tissue was assumed to have an exponential decay.^20^ A and B are the amplitude constant and rate constant for $\mu_{a}^{\text{dry}}$, respectively. This approximation was used to represent the cumulative effect of all chromophores with a Soret band in the ultraviolet-blue spectral region (e.g., collagen fibers, porphyrins) on the absorption coefficient in the visible region. The value of $f_{w}$ was fixed at 75%.^20^ The four parameters ($f_{b}$, ${\mathrm{SO}}_{2}$, A, and B) were determined by fitting the theoretical absorption coefficients calculated with this model to the absorption coefficients from each measurement. The fitting was performed via the least squares method. We obtained the changes in oxygen saturation from the first measurement to the second and third measurements along with their relationship to elapsed time.

### 2.5 Monte Carlo Simulation of Light Transport in the Lung

Using the measured optical properties, we calculated the fluence distribution and energy distribution in the lung irradiated by a 664-nm laser. These distributions were compared under different conditions: with the tumor surrounded by normal tissue versus carbon-deposited tissue, and for human lung versus porcine lung tissue. In addition, the $\mu_{a}$ values of human lung tissue were adjusted to simulate an *in vivo* oxygen saturation of 97%, and the corresponding light distribution was calculated. The calculations were performed using an MC light transport simulation.^16^ This simulation can calculate the light distribution in a complex tissue structure consisting of different types of tissues, each with its own optical properties. The light source was modeled after the side-firing probe used in a clinical trial for peripheral lung cancer.^26^^,^^27^ Light emission was assumed to be uniform from a cylindrical surface with an outer diameter of 1 mm and an emission length of 11 mm.^27^ The photon packet was set to $10^{9}$.

**Fig. 1:**
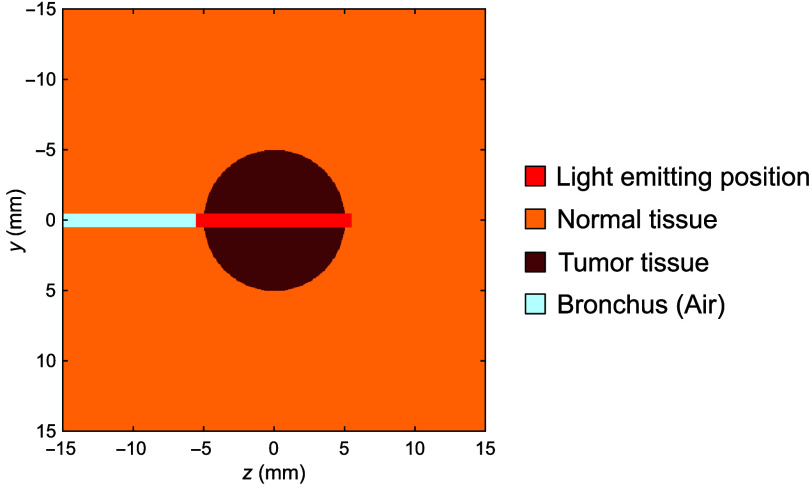
Three-dimensional numerical model of peripheral lung tissue.

A 3D numerical model of the human lung consisting of normal tissue, tumor tissue, and bronchus was developed (Fig. 1). The tumor was modeled as a 10-mm-diameter sphere centered on the bronchus, which had a diameter of 1 mm and was assumed to contain air. The computational domain was defined as 30  mm×30  mm×30  mm with a voxel size of 0.05  mm×0.05  mm×0.05  mm. The measured optical properties were assigned to each tissue type. For the air, $\mu_{a}$ was set to $1\times 10^{-5}\;{\mathrm{mm}}^{-1}$, $\mu_{s}^{\prime }$ to $0.1\;{\mathrm{mm}}^{-1}$, n to 1.0, and g to 1.0. To correct for an ${\mathrm{SO}}_{2}$ value of 97% in human lung tissue, $\mu_{a}$ was recalculated using Eq. (1) by subtracting the measured hemoglobin absorption component and adding the absorption at ${\mathrm{SO}}_{2}=97\%$. For the porcine lung model, the optical properties of tumor tissue were assumed to be identical to those of normal tissue. The tissue geometry was also assumed to be identical to that of the human lung model. The side-firing probe was positioned so that the center of its emission region was aligned with the tumor center. The irradiation power was set to 150 mW with an irradiation time of 667 s.^26^^,^^27^

## 3 Results

### 3.1 Absorption and Reduced Scattering Coefficients

**Fig. 2:**
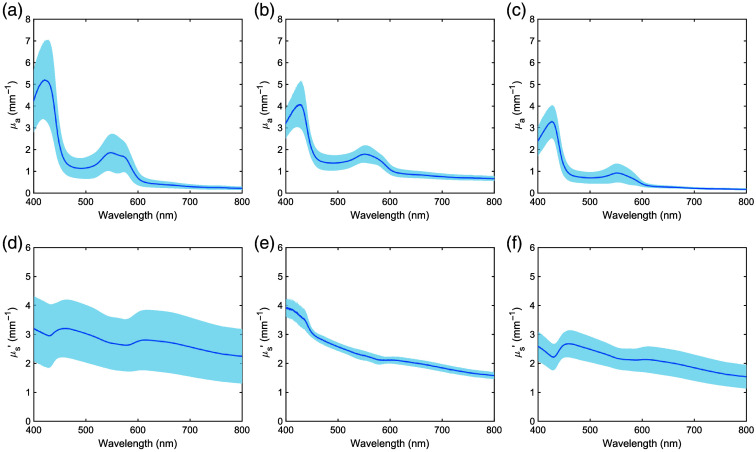
Absorption and reduced scattering spectra of [(a), (d)] normal tissue, [(b), (e)] carbon-deposited tissue, and [(c), (f)] tumor tissue of the human lung. The shaded areas represent standard deviations.

Figure 2 shows the $\mu_{a}$ and $\mu_{s}^{\prime }$ spectra of normal, carbon-deposited, and tumor tissues from human lungs. The shaded areas represent standard deviations. The sample sizes were 23 for normal tissue, three for carbon-deposited tissue, and 15 for tumor tissue. The $\mu_{a}$ spectra of normal tissue primarily showed oxygenated hemoglobin absorption peaks at 420, 548, and 575 nm. By contrast, the $\mu_{a}$ spectra of carbon-deposited and tumor tissues primarily consisted of deoxygenated hemoglobin absorption peaks at 427 and 550 nm. $\mu_{a}$ was higher for the carbon-deposited tissue than for the normal tissue beyond 600 nm and lower for the tumor tissue than for the normal tissue in 400 to 800 nm. The $\mu_{s}^{\prime }$ spectra for all tissue types showed a monotonic decrease with increasing wavelength. This trend reflects a decrease in Rayleigh scattering and an increase in Mie scattering contributions with increasing wavelength. The $\mu_{s}^{\prime }$ values were similar for all tissue types. Furthermore, there was almost no difference in the optical properties between the upper and lower lobes of the lungs (Fig. S1 in the Supplementary Material).

**Fig. 3:**
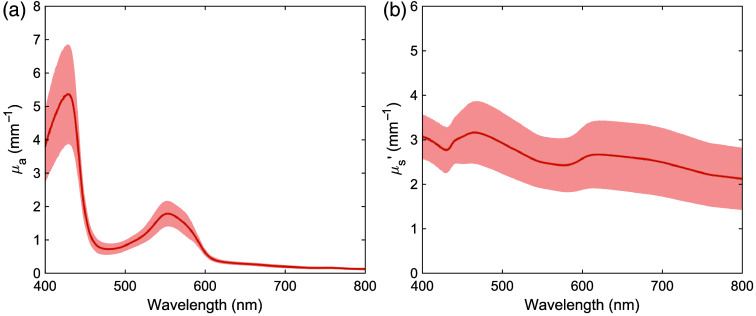
(a) Absorption and (b) reduced scattering spectra of normal tissue of porcine lung. The shaded areas represent standard deviations.

Figure 3 shows the $\mu_{a}$ and $\mu_{s}^{\prime }$ spectra of normal porcine lung tissue, with shaded areas indicating standard deviations. The sample size was 12. The $\mu_{a}$ spectra primarily consisted of deoxygenated hemoglobin absorption peaks at 429 and 553 nm. The $\mu_{s}^{\prime }$ spectra showed a monotonic decrease with increasing wavelength, similar to the trends observed in human $\mu_{s}^{\prime }$ spectra. The $\mu_{a}$ and $\mu_{s}^{\prime }$ spectra of normal porcine lung tissue were largely within the range of variation observed in the $\mu_{a}$ and $\mu_{s}^{\prime }$ spectra of normal human lung tissue. Table 2 summarizes the measured optical properties for wavelengths commonly used in PDT.

**Table 2:** List of absorption and reduced scattering coefficients of human and porcine lungs at wavelengths of 630, 635, 664, and 690 nm available for photodynamic therapy.

Tissue type	Wavelength (nm)	Human		Porcine	
		$\mu_{a}$ $\left({\mathrm{mm}}^{-1}\right)$	$\mu_{s}^{\prime }$ $\left({\mathrm{mm}}^{-1}\right)$	$\mu_{a}$ $\left({\mathrm{mm}}^{-1}\right)$	$\mu_{s}^{\prime }$ $\left[({\mathrm{mm}}^{-1}\right)$
Normal tissue	630	0.43±0.17	2.79±1.05	0.32±0.06	2.66±0.77
	635	0.42±0.17	2.78±1.05	0.31±0.05	2.65±0.77
	664	0.37±0.14	2.71±1.05	0.26±0.05	2.60±0.78
	690	0.32±0.12	2.61±1.03	0.22±0.05	2.53±0.77
Carbon-deposited tissue	630	0.89±0.18	2.06±0.13	—	—
	635	0.88±0.17	2.05±0.13	—	—
	664	0.83±0.16	1.97±0.13	—	—
	690	0.78±0.14	1.88±0.13	—	—
Tumor tissue	630	0.30±0.07	2.09±0.46	—	—
	635	0.30±0.07	2.08±0.46	—	—
	664	0.27±0.06	1.99±0.46	—	—
	690	0.24±0.05	1.89±0.45	—	—

### 3.2 Differences in Optical Properties Between Tissue Types

**Fig. 4:**
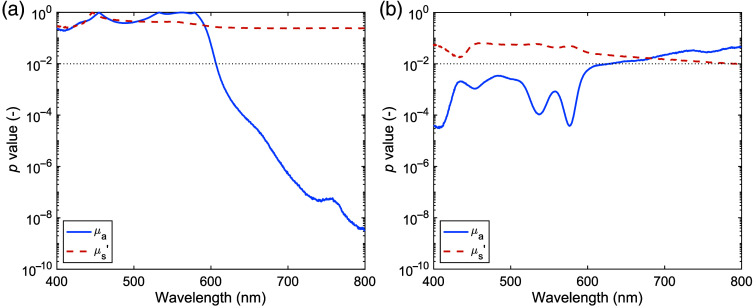
p-value plots for (a) comparison of optical properties between normal and carbon-deposited tissues of human lung and (b) comparison of optical properties between normal and tumor tissues of human lung. The solid lines represent the plot for the absorption coefficient, and the dashed lines represent the plot for the reduced scattering coefficient. The dotted lines represent the p-value of 0.01.

Figure 4 shows the p-values for comparing normal and carbon-deposited tissues and the p-values for comparing normal and tumor tissues. The $\mu_{a}$ value of carbon-deposited tissue showed significant differences from that of normal tissue at wavelengths beyond 600 nm, whereas $\mu_{s}^{\prime }$ showed no significant differences. For tumor tissue, the $\mu_{a}$ value showed significant differences from that of normal tissue in the wavelength range of 400 to 600 nm, which is strongly influenced by the absorption of oxyhemoglobin and deoxyhemoglobin. The $\mu_{s}^{\prime }$ value of tumor tissue showed no significant differences from that of normal tissue, similar to that of carbon-deposited tissue. Table 3 summarizes the four parameters obtained by fitting for normal and tumor tissues. Blood volume fraction and oxygen saturation were significantly lower in tumor tissue. The parameters related to dry tissue showed no significant differences between normal and tumor tissues.

**Table 3:** Values of $f_{b}$, ${\mathrm{SO}}_{2}$, A, and B for normal and tumor tissues of the human lung.

	This work			Bargo et al.^20^^a^	
	Normal	Tumor	p	Normal	Tumor
$f_{b}$ (%)	3.9±1.5	2.0±0.6	<0.001	3.4±1.4	9±11
${\mathrm{SO}}_{2}$ (%)	65±18	46±6	<0.001	64±18	56±24
A (${\mathrm{mm}}^{-1}$)	7±7	4±5	0.17	$(1.4\pm 2.7)\times 10^{3}$	$(1.4\pm 1.1)\times 10^{2}$
B (${\mathrm{nm}}^{-1}$)	0.004±0.001	0.004±0.001	0.33	0.011±0.007	0.010±0.002

a The data were obtained by statistically analyzing the results of *in vivo* measurements of five and six different sites for normal and tumor tissues, respectively, from two patients.

### 3.3 Oxygen Saturation Variation During Measurement

**Fig. 5:**
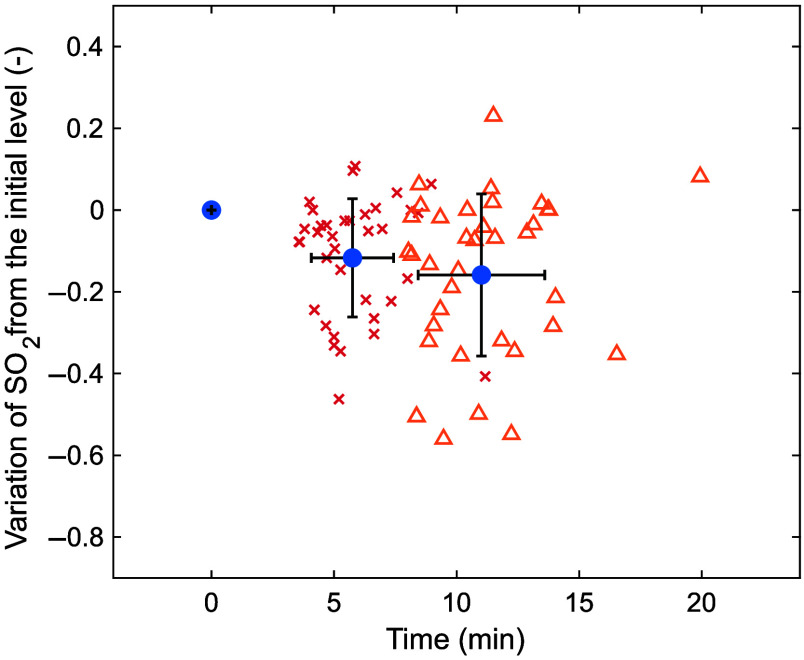
Variation in oxygen saturation from the initial level during measurement. The crosses represent values for the second measurement, and the triangles represent values for the third measurement. The circles represent the mean values for each measurement, and the error bars represent standard deviations.

Figure 5 shows the relative variation in oxygen saturation from the first measurement to the second and third measurements. Because the data show the relative variation from the first measurement, all of the measured values became zero at the first measurement. Oxygen saturation decreased on average by ∼16% from the first to the third measurement.

### 3.4 Light Distribution in the Lung

**Fig. 6:**
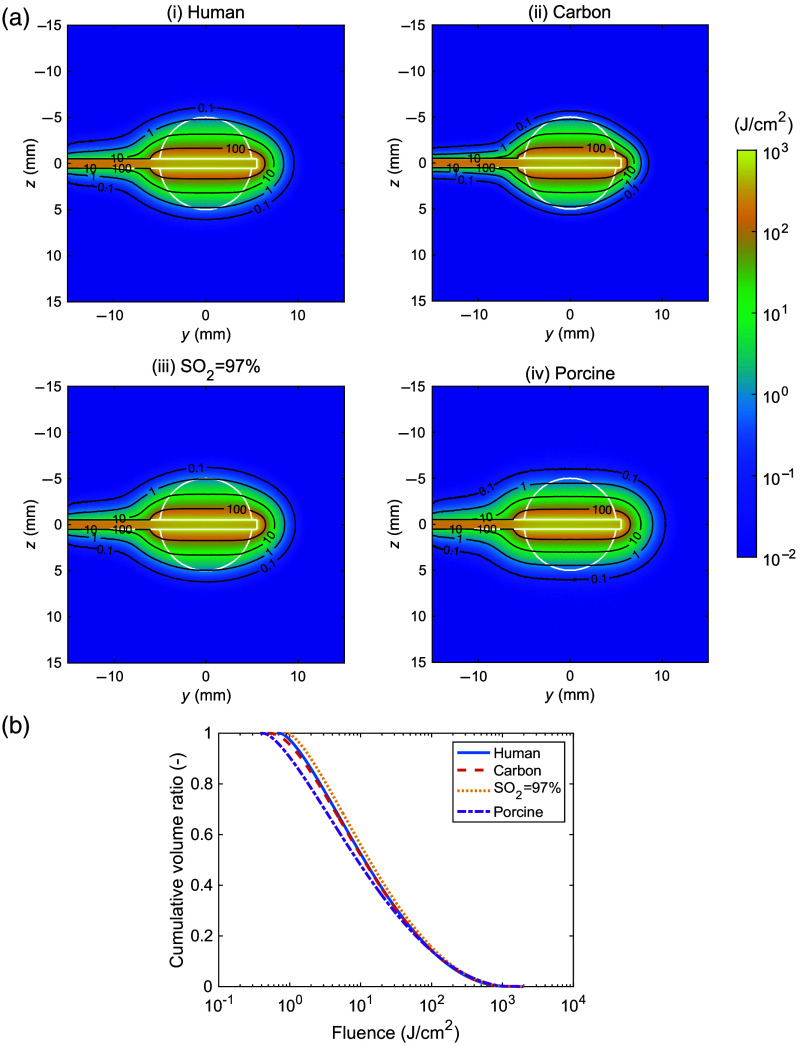
(a) Fluence distributions in (i) human lung, (ii) carbon-deposited human lung, (iii) human lung with ${\mathrm{SO}}_{2}=97\%$, and (iv) porcine lung. The white lines represent the boundary of each section shown in Fig. 1. The black lines represent fluence levels of 0.1, 1, 10, and $100\;J/{\mathrm{cm}}^{2}$. (b) Cumulative volume histograms for fluence for the tumor tissue of human lung (solid line), carbon-deposited human lung (dashed line), human lung with ${\mathrm{SO}}_{2}=97\%$ (dotted line), and porcine lung (dash-dotted line).

**Fig. 7:**
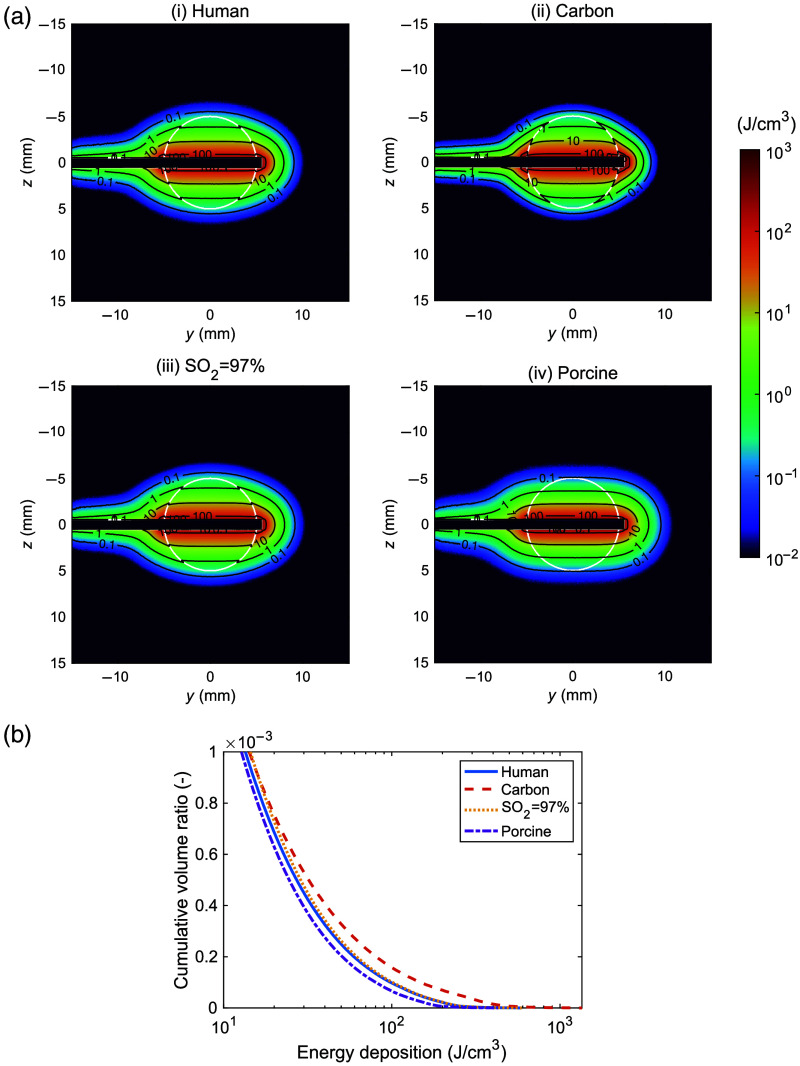
(a) Energy distributions in (i) human lung, (ii) carbon-deposited human lung, (iii) human lung with ${\mathrm{SO}}_{2}=97\%$, and (iv) porcine lung. The white lines represent the boundary of each section shown in Fig. 1. The black lines represent the energy deposition levels at 0.1, 1, 10, and $100\;J/{\mathrm{cm}}^{3}$. (b) Cumulative volume histograms for energy deposition for the surrounding tissue of the human lung (solid line), carbon-deposited human lung (dashed line), human lung with ${\mathrm{SO}}_{2}=97\%$ (dotted line), and porcine lung (dash-dotted line).

Figure 6 shows the fluence distribution under irradiation with a side-firing probe and the cumulative volume histogram of the fluence within the tumor tissue. In human lung, carbon-deposited human lung, human lung at ${\mathrm{SO}}_{2}=97\%$, and porcine lung, a 10-mm-diameter tumor was almost covered by fluence exceeding $1\;J/{\mathrm{cm}}^{2}$. The $\mu_{a}$ values for normal and tumor tissues corrected for ${\mathrm{SO}}_{2}=97\%$ were 0.35 and $0.25\;{\mathrm{mm}}^{-1}$, respectively, with the $\mu_{s}^{\prime }$ values assumed to be identical to the measured values. When carbon deposition occurred in the surrounding tissue, light diffusion into the surrounding region decreased compared with when the surrounding tissue was normal. Fluence within human tumor tissue remained largely unchanged whether the surrounding tissue was normal or carbon-deposited. Similarly, fluence within human tumor tissue showed almost no differences between the measured ${\mathrm{SO}}_{2}$ level and ${\mathrm{SO}}_{2}=97\%$. In porcine tumor tissue, the region with fluence $<1\;J/{\mathrm{cm}}^{2}$ was smaller than that in human tumor tissue, corresponding to the tumor margin. Figure 7 shows the energy distribution under irradiation with a side-firing probe and the cumulative volume histogram of energy deposition within the surrounding tissue. When the surrounding tissue contained carbon deposits, the region with energy deposition exceeding $100\;J/{\mathrm{cm}}^{3}$ was larger than that observed with normal surrounding tissue, corresponding to the region near the side-emitting probe adjacent to the tumor. At ${\mathrm{SO}}_{2}=97\%$ or in porcine lung tissue, there were almost no differences in energy deposition within the surrounding tissue compared with that in human lung tissue.

## 4 Discussion

In this study, the $\mu_{a}$ and $\mu_{s}^{\prime }$ spectra were measured for normal, carbon-deposited, and tumor tissues of human lungs in the visible to near-infrared wavelength range. The $\mu_{a}$ value of carbon-deposited tissue was significantly higher than that of normal tissue at wavelengths beyond 600 nm, which was attributed to light absorption by carbon deposits. The $\mu_{a}$ value of tumor tissue showed significant differences from that of normal tissue in the absorption bands of oxyhemoglobin and deoxyhemoglobin and with oxygen saturation. The $\mu_{s}^{\prime }$ value of normal tissue showed no significant differences from that of either carbon-deposited or tumor tissues, suggesting similar densities of scatterers such as cellular components and extracellular matrices in these tissue types.^28^ These results indicate that the differences in optical properties between human lung tissue types are primarily influenced by the degree of carbon deposition, blood content, and oxygen saturation. The variations in $\mu_{a}$ and $\mu_{s}^{\prime }$ were 40% and 37% for the normal tissue, 21% and 7% for the carbon-deposited tissue, and 30% and 21% for the tumor tissue. The tissues were obtained from nine, two, and seven patients for normal, carbon-deposited, and tumor tissues, respectively. The greater variations in optical properties observed for normal and tumor tissues are likely due to inter-patient variability.

When the $f_{b}$ and ${\mathrm{SO}}_{2}$ values obtained are compared with those reported in previous *in vivo* studies, the results are generally consistent (Table 3).^20^ The ${\mathrm{SO}}_{2}$ values were higher in normal tissue than in tumor tissue, a relationship consistent with the previous results. However, whereas $f_{b}$ was higher in normal tissue than in tumor tissue in this study, the opposite was found in previous work. Notably, the literature shows considerable variability in $f_{b}$ for tumor tissue.^20^ This variability may be due to the measurement of different human lung sites in the study, potentially influencing the reported values. The $\mu_{s}^{\prime }$ values obtained in this study were higher than those reported in the literature.^20^ The measured $\mu_{s}^{\prime }$ values reflect deflated lung tissue (i.e., intrinsic lung tissue), whereas the literature values represent *in vivo* measurements of lungs containing air. This difference in lung air content likely contributes to the observed discrepancy in $\mu_{s}^{\prime }$ because optical scattering from the lung decreases with increasing air volume.^29^ Regarding the porcine lung tissue, the $\mu_{s}^{\prime }$ at 665 nm was in good agreement with the value reported by Beek et al.^29^ and was twice as large as the value reported by Ramadan et al.^30^ The $\mu_{a}$ at 665 nm was one order of magnitude larger than those reported by the two previous studies.^29^^,^^30^ These differences may be attributed to significant differences in the anatomical and physiological characteristics of the porcine lung models examined in the previous studies and in the amount of blood at the measurement points. In addition, our experiment used double integrating sphere optics and IMC analysis, whereas the previous studies used fiber optics and the diffusion theory for the inverse analysis model. These differences in measurement system may also have influenced the variations in optical properties.^31^

In the IMC analysis, the g and n values were fixed in the 400 to 800 nm range. The fixed g value has been applied to the IMC analysis for many types of tissues, including skin tissues, brain tissues, and ureter tissues, and it has been reported that the $\mu_{a}$ and $\mu_{s}^{\prime }$ are not sensitive to g.^21^^,^^32^^–^^34^ Therefore, the assumption of g may have almost no effect on the measured $\mu_{a}$ and $\mu_{s}^{\prime }$ spectra of lung tissues. In measuring the optical properties of urinary tract tissues, sensitivity analysis was performed by changing the n value of 1.4 to 1.3 or 1.5.^21^ Although the variations in the $\mu_{a}$ and $\mu_{s}^{\prime }$ were within the standard deviation range, they were ∼20% and 10%, respectively. The assumption of the n may result in variations in the measured $\mu_{a}$ and $\mu_{s}^{\prime }$ of lung tissues.

*Ex vivo* measurements using a DIS optical system have been performed for various human tissues, including the skin,^32^ prostate,^35^ colon,^36^ liver,^37^ brain,^33^ pancreas,^38^ ureter,^21^ and lung. In this system, samples are typically sliced to a thickness of ∼1  mm, allowing optical properties to be obtained under simplified inverse analysis conditions. This method has the advantage of measuring optical properties for different tissues with consistent experimental precision and enables comparisons of optical properties between different organs. However, factors such as changes in the oxidation-reduction state and blood volume fraction can affect the *ex vivo* measurements. The observed decrease in oxygen saturation during measurements may be attributed to oxygen consumption in the blood due to cellular metabolic activity. To mitigate this effect, resected tissue should be stored in a cooled glass vial to reduce cellular metabolism.^39^ This may allow better control of the oxygenation conditions of the samples than if they are stored at room temperature. Moreover, this issue can be addressed by reconstructing the $\mu_{a}$ spectrum using the parameters in Table 3 and Eq. (1).

The results of the light propagation simulation have the potential to provide practical clinical advice on laser irradiation for PDT. One of the effective ways of determining the light dose is to perform laboratory work to estimate how the degree of necrosis changes in response to factors such as the light dose and the photosensitizer dose and then to monitor in real time with strategically placed detectors to confirm that all parts of the tumor being treated receive a light dose that exceeds the threshold.^13^^,^^40^ The numerical simulations allow the variability of the determined light dose to be evaluated by analyzing the effect of tissue characteristics such as carbon deposition and oxygen saturation, and differences between human and porcine lungs on the light distribution in the lung tissue. When carbon deposition occurred in the surrounding tissues, the fluence within the tumor tissue decreased slightly owing to the higher light absorption of carbon-deposited tissue, which reduced the contribution of backscattering from the surrounding tissues to the tumor. The energy deposition was increased in the surrounding tissue near the side-firing probe compared with the case of normal tissue. These results can be explained by a significantly higher $\mu_{a}$ in the carbon-deposited tissue than in normal tissue, and no significant difference in $\mu_{s}^{\prime }$ between the two. The fluence within the tumor tissue for the corrected $\mu_{a}$ (${\mathrm{SO}}_{2}=97\%$) was almost similar to that for the measured optical properties. This result can be explained by the fact that the difference in $\mu_{a}$ after correction was very small and included in the measurement variability for normal human tissue indicating that oxygen saturation variability has a negligible effect on the fluence in tumor tissue. This indicates that oxygen saturation variability has a negligible effect on the fluence in tumor tissue. Light distribution in the tumor tissue was largely similar between porcine and human lungs. However, the higher $\mu_{s}^{\prime }$ value in porcine tissue resulted in less light penetration and lower fluence at the tumor margin in the porcine lung than in the human lung. This suggests that using porcine lungs as a preclinical model for PDT may lead to underestimation of the light dose at the margins of a 10-mm-diameter human lung tumor. To determine whether this difference is significant, further analysis is needed to consider the individual and site variability in the $\mu_{a}$ and $\mu_{s}^{\prime }$ values. By contrast, almost no differences were observed in energy distributions within the surrounding tissue. This suggests that porcine lungs may serve as a viable model for evaluating thermal damage in the human lung. The heat derived from the energy deposition diffuses away from the target at a low fluence rate for a long duration of irradiation, but thermal damage may occur at a high fluence rate for a short duration of irradiation. These thermal effects are independent of oxygen saturation, but the biological effects associated with moderate heat are influenced by oxygen saturation. It is imperative to consider this factor when evaluating thermal damage from light irradiation in a porcine lung model.

This study has several limitations. In PDT, the lung is exposed to laser light during respiration. Although the optical properties obtained reflect the intrinsic lung tissue in a deflated state, these properties are likely to change as the alveoli expand during respiration and light is scattered along the bronchial lumen. As observed in the comparison between the *in vivo* and measured *ex vivo* optical properties, the *ex vivo* values of $\mu_{s}^{\prime }$ were larger than the *in vivo* values.^20^ In addition, changes in blood flow in the tumor tissue during laser irradiation affect the $\mu_{a}$ due to changes in volume fraction.^41^ These differences may result in increased light penetration in the real human lung compared with the simulation results. To improve the accuracy of evaluating laser irradiation for PDT, future studies will need to model optical properties that account for these factors. Another limitation is the use of a simple spherical tumor model. Although this model was used to compare the effects of the different optical properties on the light distribution in lung tissue, real tumors are often irregularly shaped, resulting in non-uniform fluence distribution, shadowing effects, and variations in local light penetration. To construct a tissue model with an irregular tumor geometry, segmented clinical images of the human lung from computed tomography and magnetic resonance imaging can be used.^30^^,^^42^ For such a tissue model, the use of MC light transport simulations is suitable because they can calculate the light distribution in a tissue consisting of different tissue types and complex structures. For future work, MC light transport simulations with a tissue model from clinical images will improve the accuracy of the light distribution in the tumor tissue, potentially providing more clinically relevant insight.

## 5 Conclusion

In this study, we measured $\mu_{a}$ and $\mu_{s}^{\prime }$ for human and porcine lung tissues *ex vivo* and evaluated the effects of carbon deposition, oxygen saturation, and differences between porcine and human lungs on the light distribution in each tissue using an MC simulation. The $\mu_{a}$ values of human lung varied between tissue types owing to the influence of carbon deposition, blood volume fraction, and oxygen saturation. By contrast, the $\mu_{s}^{\prime }$ values of the human lung showed almost no differences between tissue types. The MC simulations with the measured optical properties showed that carbon deposition in the surrounding tissue and oxygen saturation variability had almost no effects on PDT light delivery to the tumor. Moreover, although porcine lung models may underestimate the PDT light dose at the tumor margin in human lungs, they are suitable for evaluating thermal damage to the surrounding tissue. These findings provide valuable guidance for developing efficient laser irradiation protocols and critical knowledge for retrospective evaluation of treatment outcomes for laser irradiation in preclinical and clinical studies of PDT for peripheral lung cancer.

## Supplementary Material

10.1117/1.JBO.30.4.048001.s01

## Data Availability

The datasets used and/or analyzed during the current study are available from the corresponding author on reasonable request.
