# Supplementary material for: Optical characteristics of human lung cancer for photodynamic therapy with measured absorption and reduced scattering coefficients
Source: J Biomed Opt. 2025 Apr 1;30(4):048001. doi: 10.1117/1.JBO.30.4.048001 (PMC11960791; doi:10.1117/1.JBO.30.4.048001)
Supplement: Supplementary file 1 [file JBO_030_048001_SD001.docx]

**Optical characteristics of human lung cancer for photodynamic therapy with measured absorption and reduced scattering coefficients**

Yu Shimojo^1,2,3^, Yuri Morizane^2^, Takumi Sonokawa^4^, Jitsuo Usuda^4^, Takahiro Nishimura^2^

^1^ Graduate School of Medicine, Osaka Metropolitan University, Osaka, Japan

^2^ Graduate School of Engineering, Osaka University, Osaka, Japan,

^3^ Research Fellow of Japan Society for the Promotion of Science, Tokyo, Japan

^4^ Department of Thoracic Surgery, Nippon Medical School Hospital, Tokyo, Japan

**Supplementary Information**

**
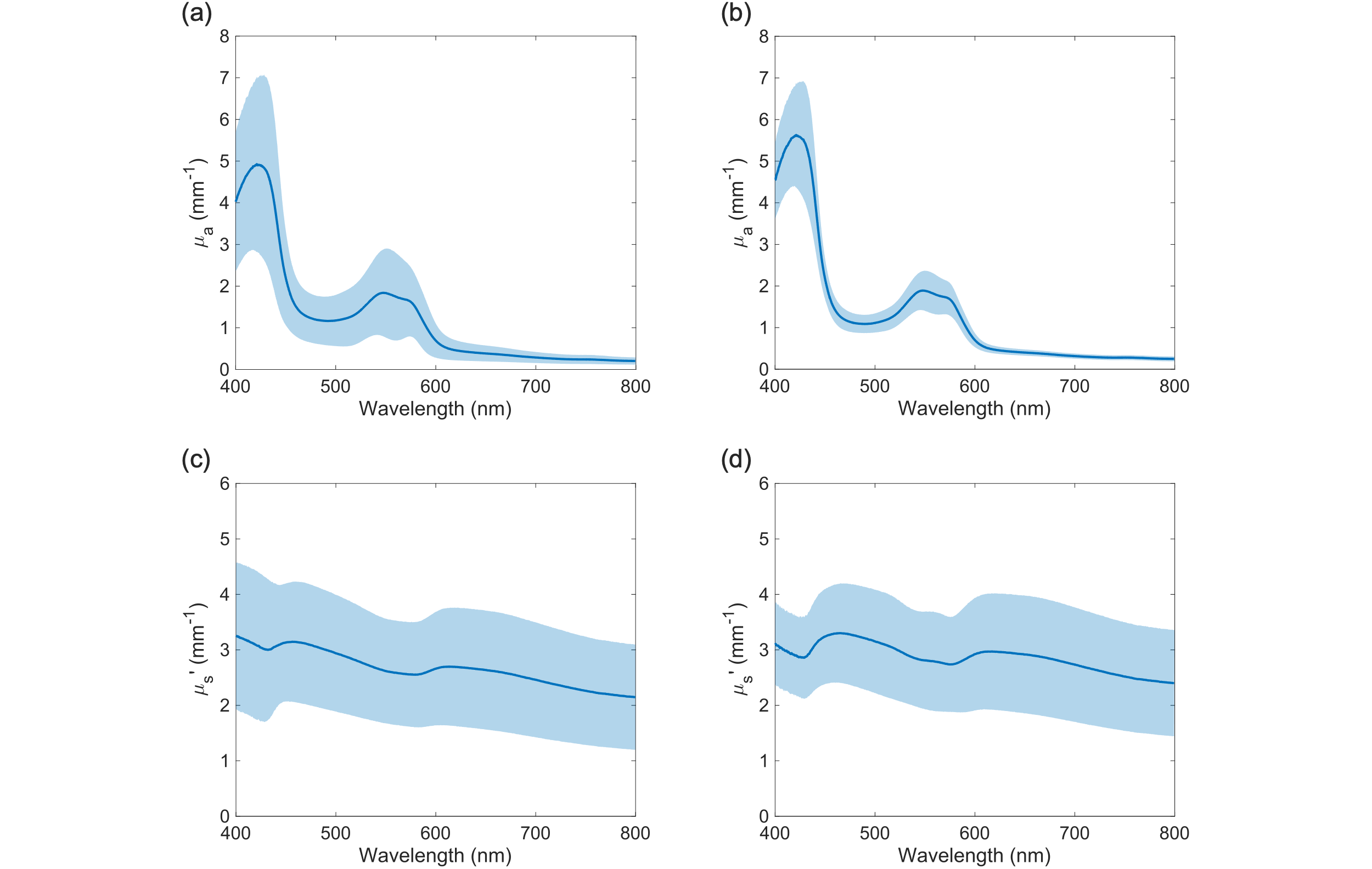
**

**Fig. S1** Absorption and reduced scattering coefficient spectra of [(a), (c)] upper and [(b), (d)] lower lobes of human lung. The shaded areas represent standard deviations.
